# The therapeutic value of treatment for multiple sclerosis: analysis of health technology assessments of three European countries

**DOI:** 10.3389/fphar.2023.1169400

**Published:** 2023-04-28

**Authors:** Lucia Gozzo, Giovanni Luca Romano, Serena Brancati, Laura Longo, Daniela Cristina Vitale, Filippo Drago

**Affiliations:** ^1^ Clinical Pharmacology Unit, Regional Pharmacovigilance Centre, University Hospital of Catania, Catania, Italy; ^2^ Department of Biomedical and Biotechnological Sciences, University of Catania, Catania, Italy; ^3^ Centre for Research and Consultancy in HTA and Drug Regulatory Affairs (CERD), University of Catania, Catania, Italy

**Keywords:** multiple sclerosis, health technology assessment, access, added therapeutic benefit, drug value

## Abstract

In accordance with European regulation, medicines containing a new active substance to treat neurodegenerative diseases as well as autoimmune and other immune dysfunctions must be approved by the European Medicines Agency (EMA) through the centralized procedure before they can be marketed. However, after EMA approval, each country is responsible for national market access, following the assessment performed by health technology assessment (HTA) bodies with regard to the therapeutic value. This study aims to provide a comparative analysis of HTA recommendations issued by three EU countries (France, Germany, and Italy) for new drugs for multiple sclerosis (MS) following EMA approval. In the reference period, we identified 11 medicines authorized in Europe for MS, including relapsing forms of MS (RMS; *n* = 4), relapsing–remitting MS (RRMS; *n* = 6), secondary progressive MS (SPMS; *n* = 1), and the primary progressive form (PPMS; *n* = 1). We found no agreement on the therapeutic value (in particular, the “added value” compared to the standard of care) of the selected drugs. Most evaluations resulted in the lowest score (“additional benefit not proven/no clinical improvement”), underlining the need for new molecules with better efficacy and safety profiles for MS, especially for some forms and clinical settings.

## Introduction

Multiple sclerosis (MS) is a chronic demyelinating autoimmune condition of the central nervous system (CNS) characterized by inflammation and neuro-axonal degeneration, leading to disease relapses and disability progression (Filippi et al., 2018; Reich et al., 2018; Brummer et al., 2022). The clinical course of the disease is variable and unpredictable in terms of both the severity and the evolution of symptoms. Most patients develop the relapsing–remitting form (RRMS), with or without permanent neurological deficits and disability (secondary progressive MS, SPMS) (Filippi et al., 2018). Moreover, a progressive disease from the onset characterizes the primary progressive form (PPMS) in some patients. The mechanisms behind the CNS damage in MS are still incompletely clarified (Ortiz et al., 2014). As an immune-mediated disease, inflammation characterizes white matter lesions, and T and B cells infiltrate the zones of demyelination, axonopathy, microglial activation, and astrogliosis (Graves et al., 2023). Inflammatory reaction can resolve despite inadequate tissue repair, resulting in astroglial scars, or become organized, fostering chronic tissue damage and remodeling (Fransen et al., 2020; Absinta et al., 2021). In patients with MS, axon and neuron injuries are closely related to inflammation but also to oxidative stress and mitochondrial dysfunction (Haider et al., 2014; Lassmann and van Horssen, 2016; Barcelos et al., 2019; Boulkrane et al., 2022).

The treatment landscape of MS has expanded very rapidly in recent years, and several therapeutic options are available for RRMS. In contrast, therapeutic alternatives for SPMS and PPMS are still limited (Faissner et al., 2019; Brummer et al., 2022).

Disease-modifying therapies (DMTs) available for the treatment of RRMS in the EU include drugs with different mechanisms of action, routes and frequencies of administration, effectiveness, and safety that are demonstrated to effectively reduce the inflammatory activity and relapse rate (Brancati et al., 2021).

Nevertheless, the efficacy of immunomodulating or immunosuppressive agents on disability progression is limited. The lack of efficacy in stopping disability progression in patients with progressive MS is due to the different underlying pathological mechanisms beyond inflammation, including CNS-intrinsic immune and degenerative processes not sufficiently targeted by the available immunomodulatory compounds (Brancati et al., 2021).

All new drugs (or all new therapeutic indications) must be authorized by a regulatory authority before they can be marketed (Gozzo et al., 2020a; Drago et al., 2020; Toro et al., 2021), and price and reimbursement procedures must be performed to find an agreement between companies and payers to guarantee market access (van Nooten et al., 2012; Gozzo et al., 2021a). In accordance with European regulation 726/2004, the great majority of new, innovative medicines pass through the centralized procedure to be marketed in the EU. This process is compulsory for human medicines containing a new active substance to treat neurodegenerative diseases as well as autoimmune and other immune dysfunctions (EMA, 2020a).

The centralized procedure allows a rapid, EU-wide authorization of medicinal products (EMA, 2020b; Gozzo et al., 2020b; Boulkrane et al., 2020; Gozzo et al., 2020c), making them available to patients throughout the EU on the basis of a single marketing authorization if the drug’s benefit–risk profile is positive according to the evidence (quality, non-clinical, and clinical data on safety and efficacy) assessment made by the European Medicines Agency (EMA).

However, each country is responsible for local market access and pricing and reimbursement decisions made according to the national health needs and resources. The assessments of medicines at the national level are made by health technology assessment (HTA) bodies, taking into account the cost-effectiveness, the therapeutic need, and the added value compared to the local standard of care (van Nooten et al., 2012; Gozzo et al., 2016; Angelis et al., 2018; Gozzo et al., 2021b; Gozzo et al., 2022).

This can result in access inequalities among European countries, based on the willingness to pay but also on the recognition of drugs’ therapeutic value (Ciani and Jommi, 2014; Akehurst et al., 2017; Allen et al., 2017).

This study aims to provide a review of the current evidence about drugs for MS approved by the EMA in recent years and to perform a comparative analysis of HTA recommendations issued by EU countries for national access.

## Methods

The study included the following steps:

1. Identification of new therapies approved for MS in Europe between January 2011 and January 2022, excluding generics and biosimilars;
2. Identification of the HTA assessments of MS drugs currently approved by EMA performed by EU national authorities in France, Germany, and Italy; selection of countries was based on the availability of assessments for public consultation and the clear definition of therapeutic values through comparable rating scales;
3. Comparative analysis of national opinions, available HTA reports, and official administrative acts of the three EU countries to compare the assessments.

Medicines centrally approved by EMA have been identified by consulting the agency’s official documents and classified by type (e.g., small molecule and monoclonal antibody), according to the orphan drug designation, and by type of authorization issued by EMA (full, conditional, and for exceptional circumstances).

The level of clinical benefit (*Service Médical Rendu*—SMR) and the added therapeutic value compared to the available therapeutic alternatives (*Amélioration du Service Médical Rendu*—ASMR) were extracted from the official HTA documentation resulting from the assessment of the Transparency Committee (TC) of the French National Authority (*Haute Autorité de santé*—HAS) (Santè, 2013; Santè, 2014).

As regards Germany, we consulted the reports of the competent national bodies (Federal Joint Committee or Gemeinsamer Bundesausschuss, G-BA and Institute for Quality and Efficiency in Healthcare, IQWIG) containing a complete HTA on the additional therapeutic benefit of the product compared to recognized standard therapies (Bundesausschuss, 2010).

Finally, we identified the therapeutic need, the added therapeutic value and the quality of the evidence from the Innovation Assessment Reports published by the Italian Medicines Agency (AIFA) (AIFA, 2017).

A direct comparison among national opinions was possible in terms of “added therapeutic value,” a measure included in all the available assessments, as reported in the Supplementary Figure S1 previously published (Gozzo et al., 2021a; Gozzo et al., 2021c).

## Results

In the reference period, we identified 11 DMTs authorized in Europe (including three monoclonal antibodies) for the relapsing form of MS (RMS, n = 4), for RRMS (*n* = 6), for SPMS (*n* = 1), and for PPMS (*n* = 1; Table 1). No drugs received a conditional approval, an approval under exceptional circumstances, or a orphan designation.

**TABLE 1:** Drugs approved in Europe in the reference period (2011–2022) for multiple sclerosis and approval details.

Active substance	ATC code	Type	Additional monitoring	Conditional approval	Exceptional circumstance	Accelerated assessment	Orphan medicine	Marketing authorization	Condition/indication
Ponesimod	L04	SM	Yes	No	No	No	No	19/05/2021	Adults with RMS with active disease defined by clinical or imaging features
Ofatumumab	L04	mAb	Yes	No	No	No	No	26/03/2021	Adults with RMS with active disease defined by clinical or imaging features
Ozanimod hydrochloride	L04	SM	Yes	No	No	No	No	20/05/2020	Adults with RRMS with active disease as defined by clinical or imaging features
Siponimod fumaric acid	L04	SM	Yes	No	No	No	No	13/01/2020	Adults with SPMS with active disease evidenced by relapses or imaging features of inflammatory activity
Ocrelizumab	L04	mAb	Yes	No	No	No	No	8/01/2018	Adults with RMS with active disease defined by clinical or imaging features
									Adults with early PPMS in terms of disease duration and level of disability and with imaging features characteristic of inflammatory activity
Cladribine	L04	SM	No	No	No	No	No	22/08/2017	Adults with highly active RMS as defined by clinical or imaging features
Peginterferon beta-1a	L03	Biologic	No	No	No	No	No	17/07/2014	Adults with RRMS.
Dimethyl fumarate	L04	SM	No	No	No	No	No	30/01/2014	Adults with RRMS.
Alemtuzumab	L04	mAb	Yes	No	No	No	No	12/09/2013	Adults with RRMS with active disease defined by clinical or imaging features
Teriflunomide	L04	SM	No	No	No	No	No	26/08/2013	Adult and pediatric patients aged 10 years and older with RRMS
Fingolimod hydrochloride	L04	SM	No	No	No	No	No	17/03/2011	As single DMT in highly active RRMS for adults and pediatric patients aged 10 years and older: with highly active disease despite a full and adequate course of treatment with at least one DMT or with rapidly evolving severe RRMS defined by two or more disabling relapses in 1 year, and with one or more gadolinium-enhancing lesions on brain MRI or a significant increase in T2 lesion load as compared to a previous recent MRI

RMS, relapsing forms of multiple sclerosis; RRMS, relapsing–remitting multiple sclerosis; SM, small molecule; SPMS, secondary progressive multiple sclerosis; PPMS, primary progressive multiple sclerosis; EDSS, expanded disability status scale; DMT, disease-modifying therapy.

Data analysis showed that for all medicines, at least one public HTA evaluation from at least one of the three selected countries was available, and for 3/11 drugs, HTA reports have been published by all three countries (Table 2). The low number of reports published by Italy is related to the fact that the assessment of innovativeness is made exclusively at the request of the pharmaceutical company.

**TABLE 2:** Agreement among opinions about therapeutic added value issued by Member States.

	Italy	France	Germany
Ponesimod	—	V^a^, HAS (2021a)	Additional benefit not proven (V)^b^/hint of a considerable additional benefit (II)^c^, (IQWIG, 2021a; IQWIG, 2022)
Ofatumumab	—	III^d^/V^e^ HAS (2021b)	—
Ozanimod hydrochloride	—	V HAS (2020c)	Low additional benefit (III)^f^/additional benefit not proven (V)^g^ (G-BA, 2021)
Siponimod fumaric acid	—	Not applicable HAS (2020b)	Additional benefit not proven (V) (G-BA, 2020)
Ocrelizumab	Low (IV)^h^ AIFA (2018a)	V^i^/III^j^/V^k^ HAS (2018)	Low additional benefit (III)^l^/additional benefit not proven(V)^m^/low additional benefit (III)^n^ (G-BA, 2018b)
Cladribine	Low (IV) AIFA (2018b)	V HAS (2020a)	Additional benefit not proven (V) (G-BA, 2018a)
Peginterferon beta-1a	—	V HAS (2015)	—
Dimethyl fumarate	—	V HAS (2014a)	Additional benefit not proven (V) (G-BA, 2016)
Alemtuzumab	—	V HAS (2016)	—
Teriflunomide	—	V HAS (2014b); HAS (2022)	Additional benefit not proven (V) (G-BA, 2014; IQWIG, 2021b)
Fingolimod hydrochloride	Important (II) AIFA (2019)	IV HAS (2019)	Additional benefit not proven (V)^o^/non-quantifiable (IV)^p^/non-quantifiable (IV) ^q^/additional benefit not proven (V)^r^ (G-BA, 2019)

^a^ Compared to ozanimod.

^b^ Adult patients with RMS with highly active disease despite DMT; comparator: alemtuzumab or fingolimod or natalizumab; adults with active RMS without prior DMT or adults with prior DMT whose disease is not highly active (EDSS >3.5); comparator: IFN-β 1a or IFN-β 1b or glatiramer acetate or dimethyl fumarate or teriflunomide or ocrelizumab.

^c^ Adults with active RMS without prior DMT or adults with prior DMT whose disease is not highly active (EDSS ≤3.5); comparator: IFN-β 1a or IFN-β 1b or glatiramer acetate or dimethyl fumarate or teriflunomide or ocrelizumab.

^d^ Compared to teriflunomide in patients with early-stage RRMS in terms of disease duration and inflammatory activity.

^e^ No clinical added value in the care pathway for patients with highly active or severe RMS in the same way as ocrelizumab.

^f^ RRMS in patients with active disease who have not previously received DMT or adult patients previously treated with DMT whose disease is not highly active; comparator: interferon beta-1a or interferon beta-1b or glatiramer acetate.

^g^ RRMS in patients with highly active disease in spite of prior treatment with DMT; comparator: alemtuzumab or fingolimod or natalizumab.

^h^ PPMS at an early stage in terms of duration of illness and level of disability, with typical radiological characteristics of inflammatory activity.

^i^ PPMS.

^j^ Versus interferon ß-1a in patients with RRMS at an early stage in terms of disease duration and inflammatory activity.

^k^ For patients with very active or severe RMS.

^l^ RMS with active disease who have not yet received DMT or adult patients with DMT whose disease is not highly active; comparator: interferon beta-1a or interferon beta-1b or glatiramer acetate.

^m^ RMS in patients with highly active disease despite treatment with DMT; comparator: alemtuzumab or fingolimod or natalizumab or, if appropriate, changes within the basic therapeutics (interferon beta-1a or interferon beta-1b or glatiramer acetate).

^n^ Early PPMS, characterized by disease duration and degree of disability, and with imaging characteristics typical of inflammatory activity; comparator: best supportive care; indication of a low additional benefit.

^o^ Children and adolescents ≥10 and <18 years of age with highly active RRMS despite treatment with at least one DMT for whom escalation of therapy is indicated; comparator: therapy according to the doctor’s instructions.

^p^ Children and adolescents ≥10 and <18 years of age with highly active RRMS despite treatment with at least one DMT for whom a change within the basic therapeutics is indicated; comparator: interferon beta-1a or interferon beta-1b or glatiramer acetate.

^q^ Children and adolescents ≥10 and <18 years of age with rapidly evolving severe RRMS defined by two or more disabling relapses in 1 year and with one or more gadolinium-enhancing lesions on brain MRI or a significant increase in T2 lesion load as compared to a recent MRI who have not yet received DMT; comparator: interferon beta-1a or interferon beta-1b or glatiramer acetate.

^r^ Children and adolescents ≥10 and <18 years of age with rapidly evolving severe RRMS defined by two or more disabling relapses in 1 year and with one or more gadolinium-enhancing lesions on brain MRI or a significant increase in T2 lesion load as compared to a recent MRI despite DMT; comparator: therapy according to the doctor’s instructions.

The highest score (“important/considerable added value”) has been recognized only for one product by Italy (fingolimod for pediatric patients aged 10 years and older with highly active RRMS disease despite a full and adequate course of treatment with at least one DMT or with rapidly evolving severe RRMS) and Germany (ponesimod in adults with active RMS disease).

Overall, 19/32 [59,3%; 10/14 (71,4%) France; 9/15 (60%) Germany] evaluations resulted in the lowest score (“additional benefit not proven/no clinical improvement”), and two Italian assessments out of three (66%) reported a “low additional benefit.”

In general, no agreements among the three EU States assessments were identified. However, the German assessment was completely in accordance with the French assessment for cladribine, dimethyl fumarate, and teriflunomide (“additional benefit not proven/no clinical improvement”).

### Ponesimod

The French authority recognized ponesimod as a first-line treatment option in active forms of RRMS. The drug demonstrated superiority versus teriflunomide in terms of reduction of the annual rate of relapses, without demonstration of superiority over reduction of the progression of disability (Kappos et al., 2021; Freedman et al., 2022). However, because robust comparative data against other medicines are not available, according to HAS the choice among the different treatments in RRMS must be made based on to the safety profile, the modes of administration, and the preferences of the patients. Moreover, because the OPTIMUM trial included patients with SPMS with superimposed relapses (Kappos et al., 2021), this population was also considered in the HTA process. However, in the absence of robust evidence, ponesimod has no place in the management of forms of SPMS in France (HAS, 2021a).

The SMR is “important” only in the treatment of adults with active forms of RRMS defined by clinical or imaging parameters and “insufficient” in other forms of MS(38). The TC considered that ponesimod does not improve the medical service provided (ASMR V), in the same way as ozanimod, in the management of active forms of RRMS(38). In contrast, Germany made a distinction according to the disease severity defined by the expanded disability status scale (EDSS) score and recognized that ponesimod offered a “hint of considerable added benefit” for adults with active RMS (without prior DMT or with prior DMT whose disease is not highly active) and an EDSS ≤3.5 (39, 40).

### Cladribine

The HTA assessments of both countries were in line for cladribine, a therapeutic option approved in patients with highly active RMS. Its efficacy has been established versus placebo in patients with predominantly not very active RRMS in terms of relapse rate and imaging criteria (Giovannoni et al., 2010; Giovannoni et al., 2018). A comparison versus other available options has been made by analyzing observational data from the CLARITY trial and an Italian multicenter database, including more than 3,000 patients who started a DMT (IFN β-1a and β-1b, glatiramer acetate, fingolimod, natalizumab, dimethyl fumarate, and teriflunomide) (Signori et al., 2020). The study showed a lower relapse rate in patients with RRMS treated with cladribine compared with matched patients treated with IFN, glatiramer acetate, or dimethyl fumarate. The effect was higher in patients with high disease activity except versus fingolimod and natalizumab (Signori et al., 2020). The data in highly active RRMS are based on *post hoc* analyses, and no data for highly active forms of SPMS are available. In the absence of direct comparison with current treatments for highly active RMS (natalizumab, fingolimod, alemtuzumab, and ocrelizumab) and due to still limited knowledge related to the safety profile, HAS considered the clinical benefit of cladribine as “low” with “no clinical added value” (V) in the management of patients with highly active RMS and recommended the use of the drug after failure of alternatives or for ineligible patients (HAS, 2020a).

For the same reason, namely, the lack of relevant data provided for the benefit assessment, the German authority granted the lowest score to cladribine (“an additional benefit is not proven”) both for patients who have not yet received DMTs or those with highly active disease despite treatment (G-BA, 2018a).

### Dimethyl fumarate

Dimethyl fumarate has not been tested in a superiority study versus an active treatment (Fox et al., 2012; Gold et al., 2012; Gold et al., 2022), even though a network meta-analysis showed a reduction in the relapse rate compared to interferon beta, glatiramer acetate, and teriflunomide. The HAS considered the indirect comparison not sufficient to draw any conclusions concerning the superior efficacy of dimethyl fumarate compared to these treatments for RRMS as well as the G-BA (HAS (2014a); G-BA (2016)).

### Siponimod

As regard to DMTs specifically approved to treat progressive forms of the disease, the efficacy and safety of siponimod were investigated in a phase III study (EXPAND trial) (Kappos et al., 2018), in participants with SPMS, of whom over 50% showed an EDSS ≥6 at study entry. Siponimod slowed the disability progression and cognitive impairment more than placebo, with an advantage in terms of relapse rate, MRI lesion activity and brain volume loss, and a safety profile comparable to that of the other drugs of the same class. Nevertheless, according to HAS, the drug has no role in the therapeutic strategy for active SPMS, taking into account the available evidence and therapeutic alternatives (HAS, 2020b). Therefore, TC considered the clinical benefit of siponimod insufficient and issued an unfavorable opinion for reimbursement.

For G-BA, the additional benefit is “not proven” for both patients with SPMS with active disease with relapses compared to interferon-beta 1a or interferon-beta 1b or ocrelizumab and patients with SPMS with active disease without relapses compared to best supportive care (G-BA, 2020).

### Ocrelizumab

As regard to DMTs approved for PPMS, ocrelizumab is recognized as a first-line treatment for patients with early-stage PPMS in terms of duration of disease and degree of disability associated with a demonstration of inflammatory activity (Ghezzi, 2018; Rae-Grant et al., 2018; Auguste et al., 2020).

In contrast, the efficacy and safety in the severe forms of PPMS have not been established, and the use should not be considered in patients with advanced disabilities (Montalban et al., 2017; Lamb, 2022).

Based on randomized clinical trials, the clinical benefit of ocrelizumab has been considered “moderate” by HAS in early-stage PPMS in terms of disease duration and degree of disability but with “no clinical added value” even in the early-stage PPMS (59). France included as rituximab as an appropriate comparator, another anti-CD20 used in PPMS even if off-label. Thus, in the lack of comparative studies versus rituximab, the role of ocrelizumab is considered unknown, and the TC recommended performing well-designed clinical trials to clarify the role of the drug in the therapeutic strategy of PPMS compared to a well-known drug, such as rituximab, that has proven to be effective and safe in this population (HAS, 2018; Brancati et al., 2021).

AIFA assigned an additional benefit of “low” (IV) for adults with PPMS at an early stage in terms of duration of illness and level of disability, with typical radiological characteristics of inflammatory activity (AIFA, 2018a). Unlike France, Italy considered the therapeutic need “maximum” due to the lack of approved treatment options. Indeed, even if the agency recognized that many off-label immunosuppressive drugs are used in clinical practice by physicians, including rituximab, no drugs are effectively approved. Then, although the magnitude of the effect on the primary outcome, the confirmed disability progression (CDP) for 12 weeks, is limited to a modest reduction compared to placebo (HR 0.76 [95% CI: 0.59, 0.98], *p* = 0.0321) (Montalban et al., 2017), the drug induced statistically significant effects in a population without authorized therapeutic alternative, but the added therapeutic benefit is considered small. Thus, AIFA restricted the use of ocrelizumab for patients according to the main inclusion criteria of the ORATORIO pivotal trial (Montalban et al., 2017; AIFA, 2022):

- 18–55 years;
- EDSS at screening from 3 to 6.5 points;
- Disease duration from onset of MS symptoms less than 15 years if EDSS greater than 5 and less than 10 years if EDSS greater than or equal to 5;
- T1 lesions G+ and/or active T2 lesions, new or expanding.

Finally, the G-BA identified best supportive care as appropriate comparative therapy and gave indication of a low additional benefit (G-BA, 2018b).

### Fingolimod

Lastly, we found disagreement about the added therapeutic value of fingolimod in pediatric patients.

Pediatric multiple sclerosis has become relatively frequent and is characterized by a high relapse rate, rapid accumulation of CNS damage, and negative long-term outcome, with a high level of physical and cognitive disability at a young age (Margoni et al., 2021).

The standard first-line DMTs for pediatric MS are interferons beta-1a/1b and glatiramer acetate, based on data collected from single- or multicenter open-label observational studies that showed their effect on clinical and MRI parameters of inflammation (Harding et al., 2013; Ghezzi et al., 2016; Baroncini et al., 2019). However, a high rate of treatment failure in response to first-line therapies has been reported, ranging from 25% to 64% (Schwartz et al., 2018), resulting in the need to switch to more aggressive DMTs.

In Italy, in adolescents aged between 12 and 18 years with rapid-changing RRMS (defined by two or more disabling relapses in 1 year and with one or more gadolinium-enhancing MRI lesions or a significant increase in the load of T2-lesions compared to a recent MRI), natalizumab may be used according to Law 648/96 as second-line treatment (Conversione, 1996). Other possible alternatives (methotrexate, azathioprine, cyclophosphamide, rituximab, alemtuzumab, and ocrelizumab) are all used off-label, and the efficacy and safety profile were not specifically studied in children and adolescents through randomized controlled clinical trials (AIFA, 2019). In this scenario, AIFA considered the therapeutic need of the pediatric population to be important.

The effectiveness and safety of fingolimod in pediatric MS were evaluated in the PARADIGM study versus IFN beta-1a, but no direct comparison has been performed versus other DMTs currently used as second-line therapies.

The drug demonstrated a significant reduction in the annualized rate of relapse compared to IFN beta-1a (RR 0.181; 95% CI 0.108–0.303; *p* < 0.001) and in other secondary endpoints (Arnold et al., 2020), including an improvement of the quality of life. Thus, in the presence of data obtained through randomized controlled clinical trials, the lack of other approved options, and the advantage of once-a-day oral administration, AIFA considered the added therapeutic value “important.”

According to HAS, the choice of first-line treatment for pediatric patients with MS should be made according to the safety profile, the route of administration, and the patient preference (HAS, 2019). In the case of highly active disease despite therapy, a more active treatment is recommended.

Fingolimod is the first product to obtain a marketing authorization in this setting for a pediatric population and to be recognized as first-line or second-line treatment for highly active forms of RRMS in pediatric patients over the age of 10 years.

Given the absence of data on the disability progression, the quality of life, and the uncertainties about the medium- and long-term tolerance, in particular related to the cardiovascular toxicity, the TC considers that fingolimod brings “minor improvement” of the medical service rendered (ASMR IV) for highly active forms MS in pediatric patients aged 10–18 years.

G-BA distinguished several situations among the general pediatric approved indication (G-BA, 2019):

1. Children and adolescents ≥10 and <18 years with highly active RRMS despite treatment with at least one DMT for whom escalation of therapy is indicated;
2. Children and adolescents ≥10 and <18 years with highly active RRMS despite treatment with at least one DMT, for whom a change within the basic therapeutics is indicated;
3. Children and adolescents ≥10 and <18 years with rapidly evolving severe RRMS defined by two or more disabling relapses in 1 year and with one or more gadolinium-enhancing lesions on MRI or a significant increase in T2 lesion load as compared to a recent exam who have not yet received DMTs;
4. Children and adolescents ≥10 and <18 years with rapidly evolving severe RRMS defined by two or more disabling relapses in 1 year and with one or more gadolinium-enhancing lesions on MRI or a significant increase in T2 lesion load as compared to a recent exam despite DMT.

The German authority issued an opinion for each clinical situations; in particular, “an additional benefit not proven” for 1 and 4 and “hint for a non-quantifiable additional benefit” for 2 and 3.

## Discussion

In accordance with European regulations, medicines containing new active substances to treat neurodegenerative diseases as well as autoimmune and other immune dysfunctions must be approved by the centralized procedure before they can be marketed in Europe. After EMA approval, each national HTA body is involved in decisions about market access, following the assessment of the risk–benefit profile and the comparative therapeutic value.

In this study, we selected medicines recently approved by EMA which represent potential innovative treatment for MS. Our results showed a lack of agreement among EU national authorities about the therapeutic value (in particular the “added value”) of drugs recently approved for different forms of MS. Overall, the opinions issued by national authorities were negative because the added therapeutic value has been classified as “not proven” in 19 out of 32 (59.3%) assessments, in particular in France (10/14; 71.4%), underlining that the unmet medical need for MS, especially for some forms and clinical settings, is still high, and new molecules with better efficacy and safety profile are expected.

In line with this demand, several clinical trials are ongoing, as detected on clinicaltrials.gov (Table 3, update February 2023). In general, progressive forms of MS represent a high unmet need because therapies that convincingly affect progression in these patients have yet to be identified (Metz and Liu, 2018), due to poor characterization of the pathological processes behind progression, the lack of good animal models, and absence of validated surrogate endpoints.

**TABLE 3:** Ongoing clinical trials available on *clinicaltrials.gov* for multiple sclerosis (MS) as “condition or disease,” including only those with the following status: “not yet recruiting,” “recruiting,” “enrolling by invitation,” “active, not recruiting,” and excluding those without therapeutic intervention (update February 2023).

Indication	No. of studies
MS (overall)	467
RRMS	76
PPMS	22
SPMS	27
Funded by companies	150
Phase I	20
Phase II	33
Phase III	59
Phase IV	27
Other funders	322
Phase I	33
Phase II	40
Phase III	14
Phase IV	13
Including children/adolescents	28
Funded by companies	11

Inflammation is certainly part of the process but the anti-inflammatory DMTs available for RMS can control at most only the relapse-related disability progression (Hughes et al., 2018). Drugs targeting the other pathological features of progression, such as demyelination, axonal loss, mitochondrial dysfunction, and neurodegeneration, are still not available.

Neurodegeneration may be related to loss of myelin-protective functions, abnormalities of blood–brain barrier (BBB), and also to dysregulation of function of glial cells, including microglia (Collongues et al., 2022). Neuroprotection can be achieved by different mechanisms of action, including the regulation of axonal function, glial function, BBB integrity, and myelin-protective function.

In this context, we found ongoing studies with Bruton’s tyrosine kinase inhibitors (BTKi), which act on key pathways regulating activation, proliferation, survival, and differentiation of B cells and other immune cells and also microglia (Carnero Contentti and Correale, 2020). The modulation of microglial activity by BTKi can be useful in MS suppressing inflammation and in supporting remyelination (Martin et al., 2020; Geladaris et al., 2021). Tolebrutinib and fenebrutinib reached relevant concentration in the CNS and currently are under evaluation in phase III clinical trials (PERSEUS-NCT04458051 in PPMS, HERCULES-NCT04411641 in SPMS comparing tolebrutinib versus placebo, and FENtrepid-NCT04544449 in PPMS comparing fenobrutinib versus ocrelizumab) (Reich et al., 2021; Collongues et al., 2022; Schneider and Oh, 2022).

The therapeutic added value of new drugs versus available treatments is one of the main points of the HTA process. In line with previous observation, we found heterogeneity of the HTA opinion issued by Member States, while relying on the evaluation of the same clinical data (Gozzo et al., 2021a; Gozzo et al., 2021c). This heterogeneity does not necessarily translate into different reimbursement decisions but can determine different eligibility criteria among countries resulting in variable patient access.

A critical point in the comparative analysis typical of the HTA process is the choice of an appropriate comparator.

A comparator in a relative effectiveness assessment (REA) is a technology or an intervention with which compare the new technology in order to establish its added therapeutic value (EUnetHTA, 2015; Commission, 2017). In general, the appropriate comparator should be used in the routine clinical practice in the individual healthcare system according to updated European or international guidelines and be approved by regulatory authorities for the appropriate indication. However, there is no consensus across European countries on the definition of routine clinical practice; moreover, sometimes the choice of comparator is controlled by law and can take into account the cost of treatment. The definition of standard of care is facilitated only for rare diseases, in particular for the lack of therapeutic options. Our previous study about the alignment of HTA assessments for advanced therapeutic medicinal products (ATMPs), mostly developed for rare diseases, showed a low rate of agreement on the therapeutic value of ATMPs approved in Europe (Gozzo et al., 2021a). In this case, the choice of comparator was not a critical variable due to the lack of alternatives in most of the indications or the availability of one single comparator.

In contrast, in the current study, we found some critical issues in this area. For example, France identified rituximab, an anti-CD20 that demonstrated effectiveness and safety in patients with PPMS, as appropriate comparator to evaluate the value of ocrelizumab (HAS, 2018; Brancati et al., 2021). In the lack of direct comparison, the role of ocrelizumab remains unknown, and France recommended performing randomized clinical trials versus rituximab to clarify the value of the drug in this population. Meanwhile, Italy and Germany did not include rituximab in the analysis, and considered the therapeutic need in this setting as “maximum,” due to the lack of approved treatment options, even if many off-label immunosuppressants are used in routine clinical practice. It is noteworthy that a non-inferiority phase III study (the DanNORMS trial-NCT04688788, promoted by a Danish hospital) directly comparing ocrelizumab and rituximab in active MS, including progressive MS, is currently ongoing, with an estimated completion date in 2028.

The lack of direct comparisons, in particular versus other second-line DMTs, probably affected also the assessment of fingolimod in the pediatric population.

It is hoped that the adoption of the new regulation on HTA with the aim to harmonize HTA methodologies in Europe will reduce disparities of assessment of medicines among European countries (Lucia Gozzo et al., 2022).

Unfortunately, the joint clinical assessment reports do not include the overall benefit, nor the added clinical value, and Member States are still solely responsible for national HTA processes for the definition of the therapeutic added value. Moreover, the new regulation does not guarantee overcoming this critical issue as regard to the selection of comparator in order to align the HTA approaches (Julian et al., 2022).

Therefore, the new regulation will probably represent a missed opportunity to unify the HTA in EU and ensure rapid and uniform access to innovation for patients who can benefit.

## Data Availability

The original contributions presented in the study are included in the article; further inquiries can be directed to the corresponding author.
